# Molecular medicine-based IBD treatment strategies—we take it personally!

**DOI:** 10.3389/fgstr.2023.1226048

**Published:** 2023-08-09

**Authors:** Viktoria Hentschel, Jochen Klaus

**Affiliations:** Department of Internal Medicine I, University Clinic Ulm, Ulm, Germany

**Keywords:** personalized medicine (PM), Crohn’s disease, ulcerative colitis, biomarker, prediction of response to therapy

## Abstract

In light of potentially aggressive disease courses of either IBD type—CD or UC—marked by frequent flareups or non-subsiding inflammatory activity, effective immunosuppression is key to preventing progressive tissue destruction and permanent disability. However, over-treating patients with a high probability of an indolent disease course ought to be avoided. To solve this therapeutic dichotomy, there is a pressing need for a reliable classification of patients based on their biosignature to rate their individual prognosis and likelihood of response to a given therapy. This need for pinpoint therapeutic strategies is addressed by the concepts of PreM and the more stringently defined PerM. In this review we summarize the most pivotal study results published so far in the field of individualized IBD care with a special focus on molecular diagnostics and their applicability in the clinical setting.

## Introduction

In recent years, the pharmacological management of CD and UC has seen impressive advances in the development of targeted therapies, resulting in the definite or imminent approval of several cutting-edge immunomodulatory agents. Despite the diversification of available treatment options, it remains a challenge for clinicians to anticipate a patient's long-term disease course upon first diagnosis, and thus to determine with certainty *a priori* which patients will be at high risk of complications. Furthermore, to date, there is no panel of readily applicable biomarkers that are predictive of which cytokin-driven signaling pathway contributes to the inflammatory process the most and therefore should be addressed with priority. As a consequence, patients undergo uniform therapy schedules, usually including an anti-TNF-α or anti-IL12/23 inhibitor as first-line induction that, in placebo-controlled trials, have shown statistically significant yet clinically mediocre response rates (1, 2).

The first abovementioned statement addresses the need to estimate the treatment intensity required to induce satisfactory disease control, which in clinical routine is usually guided by demographic and clinical features. Data from a retrospective multi-center study suggest that a younger age at disease onset, male gender, and refractoriness to steroids portend a significantly higher risk for a prognostically unfavorable course of CD, warranting the administration of immunosuppressive therapy (3). Similarly, a comprehensive literature review embedded in the IBD Ahead 2014 educational initiative identified young age at diagnosis, extensive involvement of the gastrointestinal tract, deep ulcerations at index endoscopy, perianal/rectal disease, and penetrating/stricturing behavior as being associated with the highest probability of developing complications at a later stage in patients with CD (4).

The second statement underscores the importance of selecting a biological agent that the patient will most likely benefit from. Clinical experience suggests that the first hit is most critical, as response rates of TNF-α-experienced patients who need to be switched to another anti-TNF-α agent due to primary or secondary failure rapidly decline with every additional line of treatment, even if agents with a different mechanism of action are subsequently used (5–8). Furthermore, a biological agent’s effectiveness may depend on the timing of its first-time application, with biological therapies starting within 18 months of disease duration resulting in higher rates of remission induction (9).

In an ideal setting, clinical decision-making results in the avoidance of both inappropriately deep immunosuppression and the administration of biological agents with foreseeably limited efficacy, while at the same time targeting the most detrimental pro-inflammatory signaling cascade with an immunomodulatory agent at the lowest possible dosage.

This therapeutic challenge has promoted the ascent of precision medicine (PreM) and personalized medicine (PerM). In PreM, patients are grouped into subpopulations according to specific genetic, phenotypic, environmental, and lifestyle traits with a significant impact on disease progression, which guide the choice of treatment they receive (10, 11). By contrast, PerM refers to an individualized approach, utilizing a patient’s information to predict their individual disease course and select the most promising treatment option (12). To date, both concepts barely play a role in clinical reality, and this is especially true for PerM.

**Figure 1:**
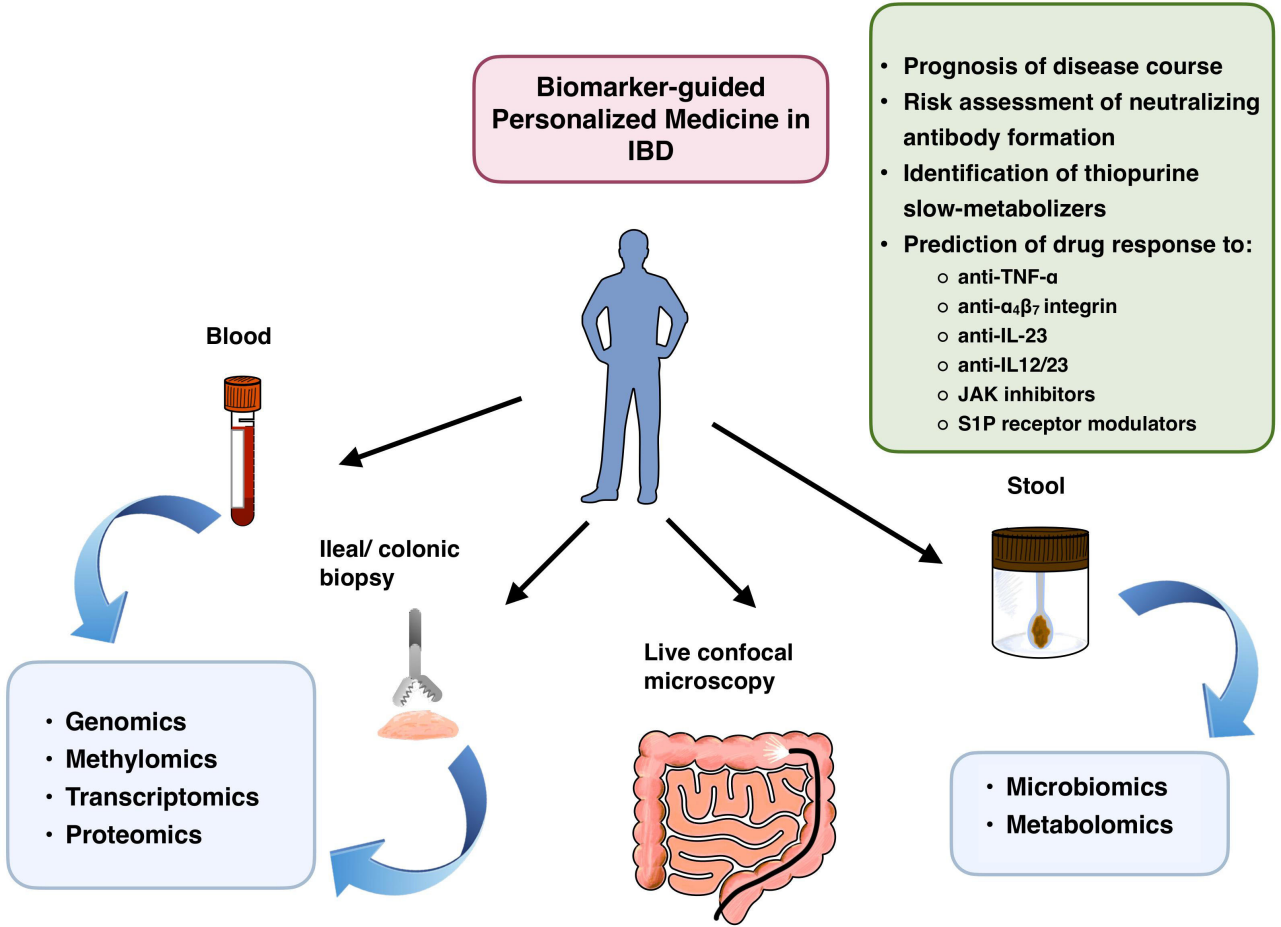
Potential fields of PerM application in patients with IBD, stratified by bioresources and molecular analytical methods. JAK, janus kinase; S1P, sphingosine 1-phosphate.

Upon *ex ante* use of PerM, healthcare providers are uncertain about how the patient’s condition will evolve in the future, and therefore rely on existing evidence to take therapeutic action based on the patient’s biological signature at baseline. Conversely, the *ex post* approach is characterized by a retrospective interpretation of data, linking biomarker analysis results with the actual clinical outcome following initiation of therapy. Unlike more comprehensive reviews such as that (13) recently published by Vieujean et al., which covers a variety of diagnostic features applicable to PerM, this mini-review briefly summarizes the latest study results related to promising candidate biomarkers according to their molecular targets and potential clinical impact, and finally, provides a glance at the prerequisites of successful implementation of molecular profiling as a mainstay of PerM in real-world IBD care. Available sources of biomaterials, analytical approaches, and potential implications of the results obtained are displayed in **Figure 1**.

## Foretelling the future

Prognostic prediction models, which otherwise are exclusively composed of clinical and serologic parameters, can be substantially enriched by the addition of molecular markers. In addition to genetic variants indicative of disease susceptibility such as the IL23R SNP rs1004819 G > A and rs7517847 T > G (14), genome-wide association studies have unveiled several prognostic loci, including NOD2 (15, 16), IRGM (17), and ATG16L1 (18), which seemingly confer a higher risk of progression to surgery in CD patients. However, in *post hoc* analyses, this association was found to be confounded due to a preferred affection of the ileal segment, for which ileocecal resection is a commonly performed surgical intervention (19, 20). Another study identified FOXOS, XACT, a region upstream of IFFBP1, and the MHC region as four loci of genome-wide significance that were exclusively associated with prognosis but, remarkably, not with disease susceptibility, pointing to the existence of distinct susceptibility and non-susceptibility variants (21).

In a multi-center pediatric cohort with a first diagnosis of CD, stricturing behavior was significantly associated with a pro-fibrotic gene expression pattern in ileal biopsies, resulting in improved overall performance of the study’s competing risk model (22). Since it is more convenient to conduct in daily practice, a whole blood-based transcriptomic assay on a set of 17 CD8^+^ T-cell genes (PredictSure IBD™) to distinguish between potentially severe and mild courses of newly diagnosed CD and UC has been made commercially available (23). Translated to clinical practice, both study findings allow the risk-stratified identification of patients likely to benefit from early anti-TNF-α therapy to avoid penetrating CD (22) or generally to require a more aggressive “top-down” rather than a more hesitant “step-up” treatment approach (23).

If a patient’s condition mandates implementation of a therapy with a biologic, it cannot be determined with certainty which agent will prove most advantageous in terms of swift and sustainable disease control. The latter requirement may be compromised by the production of neutralizing anti-drug antibodies, which do not only depend on the agent’s inherent immunogenicity but also on the patient’s individual genetic susceptibility to antigen recognition. A significantly increased risk of developing antibodies against the anti-TNF-α agents infliximab and adalimumab was documented in CD patients carrying the HLA-DQA1*05 allele (24). Positive genotyping ahead of a planned anti-TNF-α treatment may therefore provide a strong argument in favor of adding an immunomodulator such as azathioprine or mercaptopurine. Since thiopurines confer a dose-dependent risk of myelosuppression, regular monitoring of the patient’s complete blood count to rule out thiopurine-induced leukopenia is mandatory. Several reports emphasize the potential implications of various polymorphisms of thiopurine S-methyltransferase (TPMT) and nudix hydrolase-15 (NUDT15), which impair the metabolism of biologically active 6-thioguanine and cause lymphocyte apoptosis by incorporation of thio-nucleotides into newly-synthesized nucleic acids, respectively (25). In particular, the NUDT15 SNPs c.415 C>T (26) and p.Arg139Cys (27) detected in IBD patient populations from India and Korea were found to be associated with odds ratios ranging between 3.6 and 35.6 for the development of thiopurine-induced leukopenia. This finding was replicated in a subsequent genome-wide association study on IBD patients of European descent, which discovered three additional NUDT15 variants (28). These findings illustrate that the implementation of screening for clinically relevant TPMT and NUDT15 variants allows preventive dose adjustment of thiopurines, thereby enhancing the safety of a concomitant immunomodulator therapy.

## Prediction of drug response

### TNF-α signaling

Being the most frequently administered class of biologic agents, several studies have investigated potential biomarkers predicting response to anti-TNF-α agents. Lipopolysaccharide-stimulated peripheral blood mononuclear cells release higher amounts of TNF-α if derived from CD patients with a clinical response to infliximab, delineating primary responders with 100% sensitivity and 82% specificity at a cutoff value of 500 pg/mL (29). TNFR2 expression on intestinal CD3^+^ T cells was significantly elevated in anti-TNF-α-naive CD patients who later proved to be anti-TNF-α responders (30). In an attempt to identify a valid set of *ex-ante* biomarkers predictive of anti-TNF-α response, Mishra et al. opted for blood-based multi-omic analysis across 14 weeks following therapy induction with infliximab. Although the study failed to detect robust baseline signatures for therapy response, gene network construction revealed transcriptional modules involved in type I interferon signaling pathways, platelet aggregation, and erythropoiesis, which were subject to early disruption in TNF-α responders but preserved in non-responders (31). This finding could be exploited for early assessment of response following the empirical one-time administration of infliximab, thus sparing the patient a futile immunosuppressive treatment.

Low mucosal and, to a lesser degree, serum expression of TREM1 was found to be indicative of long-term anti-TNF-α-induced endoscopic remission (32). By contrast, increased mucosal tissue levels of oncostatin M and its receptor are associated with increased histopathological disease activity and an early need for biologics and may be predictive of a primary endoscopic non-response to both anti-TNF-α agents and vedolizumab (33–35).

Direct inspection of the epithelial lining for membrane-bound TNF-α as a mucosal predictor of anti-TNF-α response was enabled by live confocal imaging after endoscopically applying fluorescent-labeled adalimumab with a spray catheter; IBD patients with ≥ 20 positive cells per confocal image achieved a response rate of 92% while patients under this cell count had only a 15% chance of response (36). Despite providing an alluring method of gaining immediate insight into mucosal immunoreactivity toward anti-TNF-α agents, this prediction tool comes with considerable technical expenditures and invasiveness to the patient, making it impractical for frequent patient screens in clinical routines.

The bacterial ecosystem is susceptible to uncontrolled IBD activity, resulting in a shift of microbiota composition, diversity, and metabolic dysfunction. Metabolic network remodeling based on anti-TNF-α pre- and post-treatment fecal samples revealed that non-remitting patients displayed a reduction of total metabolic exchange already at baseline, whereas the remitting patients’ metabolic profile was not significantly altered compared with healthy controls. *In silico*-generated findings were subsequently confirmed by fecal metabolomic analysis in a validation cohort, pointing to a potential role for the microbial metabolome in identifying future anti-TNF-α responders (37).

### α_4_β_7_ signaling

The principal mode of action of the monoclonal antibody vedolizumab (VDZ) involves reducing leukocyte infiltration of the intestinal mucosa by impeding α_4_β_7_ integrin-mediated transendothelial migration. Not surprisingly, pretreatment α_4_β_7_ expression in multiple subsets of T, B, and natural killer cells, in addition to serum trough levels, are positively correlated with clinical response to VDZ. By contrast, a high proportion of effector memory T cells with unsaturated α_4_β_7_ binding sites indicates that chances of a VDZ response are lower (38). Based on RNA sequencing of inflamed colonic biopsies at baseline, endoscopic VDZ remitters reproducibly exhibit a four-gene expression signature comprising the genes *PIWIL1*, *MAATS1*, *RGS13*, and *DCHS2*, which were found to be significantly upregulated compared with non-remitters (39). However, it remains inconclusive how this group of genes, with largely ill-defined or unknown functions, is linked to VDZ efficacy. As outlined in the previous section “TNF-α signaling”, the same group headed by Atreya applied *in vivo* molecular imaging in five patients with TNF-α refractory CD, using fluorescent-labeled α_4_β_7_ antibodies to quantify α_4_β_7_ expressing mucosal cells. Although limited to only five patients, this technique reliably distinguished between α_4_β_7_ probe-positive VDZ responders and probe-negative VDZ non-responders (40).

Another group carried out serum cytokine arrays in UC patients newly started on VDZ, finding baseline IL-8 serum levels and the net decline of IL-6 and IL-8 serum levels over 6 weeks to be significantly correlated with endoscopically scored mucosal healing at week 54 (41). However, due to a small number of patients (*n* = 27), study results do not allow the drawing of firm conclusions on the long-term predictive power of single-cytokine values. In addition, it would have been of interest to assess intestinal biopsies for tissue cytokine levels and grade inflammation histologically to verify deep mucosal healing.

In addition to acquiring predictive data pertinent to the immune system or mucosal gene expression, metagenomic stool microbiome analysis in patients with CD colitis prior to initiation of VDZ therapy can be used to rate the chances of clinical remission at week 14. Linking microbiota with the respective clinical phenotype at each follow-up visit allowed researchers to build a network algorithm termed vedoNet to enable the prediction of response to an anti-integrin therapy. In this study, increased baseline abundance of *Roseburia inulinivorans* and *Burkholderiales* species, and pathways involved in branched-chain amino acid synthesis, including L-citrulline and L-isoleucine, were noted to be significantly associated with week 14 remission (42). Despite the recent emergence of artificial intelligence-assisted high-throughput strain isolation and genotyping platforms, facilitating systematic microbiome sequencing and biobanking of human stool samples (43), metagenomic analysis results derived from complex microbial communities should always be interpreted in conjunction with dietary factors and correlated with objective criteria of response, followed by validation in larger patient cohorts, all of which were major limitations acknowledged by the authors of the abovementioned study.

### IL-23/IL-22 signaling

Accumulating evidence suggests a pivotal role for IL-23-related signaling in the pathogenesis of CD [for more detailed information, we kindly refer the reader to a comprehensive review by (44)]. Ustekinumab (UST) is the first human monoclonal antibody directed against the shared p40 subunit of IL-12- and IL-23 that was approved for the treatment of CD and UC (45). Based on the results from the phase 3 ADVANCE and MOTIVATE induction trials, the first selective IL-23 inhibitor risankizumab has recently been approved for the treatment of moderate-to-severe CD with a contraindication or failing to respond to both conventional and anti-TNF-α therapy (46). Other IL-23 inhibitors such as brazikumab, mirikizumab, and guselkumab are currently under investigation, demonstrating promising preliminary results in advanced-stage clinical trials (47, 48).

In a prospective study, low mucosal IL-23A expression at baseline was found to be strongly associated with resistance to UST (49). In another study, TNF-α-refractory CD patients displayed mucosal expansion of a population of apoptosis-resistant IL23R^+^ CD4^+^ T cells, which was dependent on paracrine IL-23 signaling from CD14^+^ macrophages (30). Single-cell transcriptomic analysis of *ex vivo*-stimulated IL-23-producing human monocytes revealed several hyperinflammatory clusters resembling the previously reported signature of inflammatory mononuclear phagocytes from CD and UC patients. Correlation with ileal tissue gene expression data derived from the RISK CD cohort (22) finally identified a subset of monocyte genes that were associated with IL-23-mediated intestinal inflammation and predicted TNF-α non-response in both CD and UC patients (50). Based on these results, the detection of such a pathogenic signature may specifically warrant anti-IL-23p19 inhibition, optionally augmented by blocking upstream regulation *via* IL-1.

Pharmacological IL-23 blockade implicates co-inhibition of the downstream-regulated cytokine IL-22, which has an ambiguous role with respect to intestinal epithelial barrier recovery and perpetuation of chronic inflammation (51, 52). Only recently, a study by Pavlidis et al. has shed light on the clinical and functional relevance of the IL-22 responsive transcriptional program in the diseased tissue of UC patients treated with UST. A central finding of this study was that IL-22 acts as a major regulator of neutrophil recruitment to the colon by driving the expression of neutrophil-active CXC-family chemokines, which in turn is associated with refractoriness to UST (53). The molecular typing of patients based on their transcriptional profile may therefore help in differentiating between potential UST responders and non-responders at an early stage.

## A leading part for biomarkers?

To date, IBD treatment is still largely determined by error-prone stochastic considerations related to the patient’s clinical phenotype and the healthcare provider’s personal experience. However, the outstanding socioeconomic importance of IBD care demands a multilayered approach, taking into account not only disease activity, distribution and extent of the inflammatory burden, and the presence of extraintestinal manifestations, but also field-tested, highly specific molecular biomarkers from easily accessible sources. The ongoing search for potential biomarkers is supported by a growing number of large-scale prospective research studies, aiming at merging clinical information on the patient’s individual drug response and environmental and dietary elements with genomic, transcriptomic, proteomic, and microbial data sets to create comprehensive multi-omics repositories (22, 54–56). Such integrative data collections can, for instance, be utilized to unravel potential mechanisms of interaction between microbial proteins and host gene expression or to simultaneously trace longitudinal changes in the metagenomic, metatranscriptomic, and metabolomic profile of the microbiomes of IBD patients (55, 57). With the increasing need for high-dimensional data processing, machine-learning approaches have steadily gained importance as a means of facilitating big-data handling. By applying supervised machine-learning algorithms, co-dependencies between known input and output variables can be uncovered, and unsupervised learning algorithms can enable the visualization of latent patterns by dimensionality reduction. The usefulness of machine learning-based personalized approaches in the field of IBD has already been elaborated in a review by Stankovic et al. to which we kindly refer the reader of this article (58).

If placed at the scientific community’s disposal, those large databases may serve as appropriately powered validation cohorts for smaller pilot studies, paving the way toward the discovery and corroboration of novel biomarkers, ideally specific to the IBD subtypes CD and UC. It is much hoped that patients’ individual molecular footprints will at some point attain the overarching goal of PerM to become a decisive factor in the choice between different treatment strategies, ensuring the most favorable outcome at reasonable costs.

## Author contributions

VH and JK contributed to the conception and structure of the review. VH wrote the first draft of the manuscript. JK revised the manuscript. All authors contributed to the article and approved the submitted version.
